# Supplementary material for: NASC-seq monitors RNA synthesis in single cells
Source: Nat Commun. 2019 Jul 17;10:3138. doi: 10.1038/s41467-019-11028-9 (PMC6637240; doi:10.1038/s41467-019-11028-9)
Supplement: Supplementary file 1 — Supplementary Information [file 41467_2019_11028_MOESM1_ESM.pdf]

Supplementary Information

NASC-seq monitors RNA synthesis in single cells

Hendriks et al.

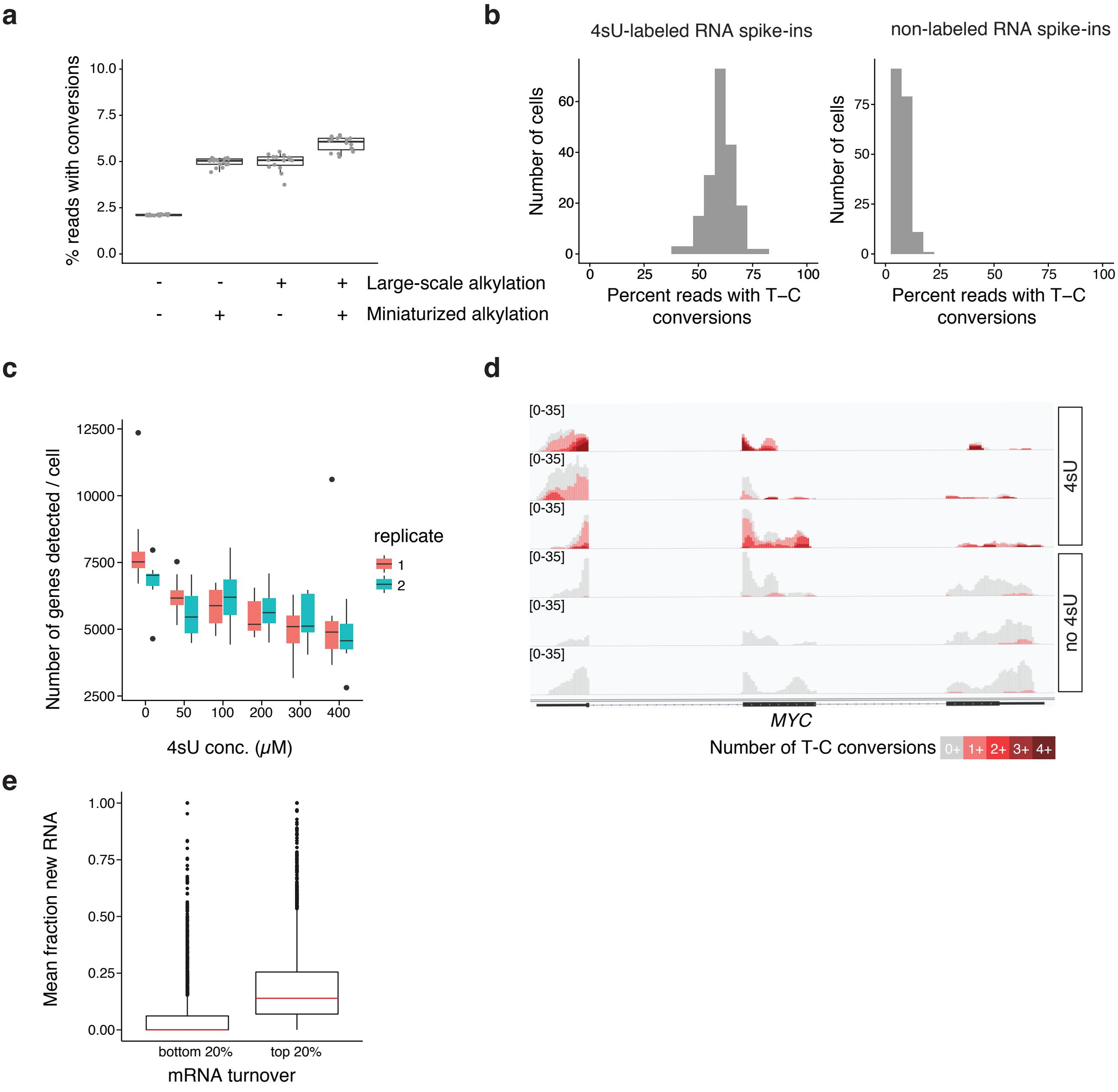

Supplementary Figure 1. Detailed characterization of NASC-seq on K562 cells.

(a) Comparison of bulk and low-input alkylation. 50 pg of bulk RNA from K562 cells, was labelled for 3h with 500 µM 4sU and subjected to alkylation either during the NASC-seq protocol (miniaturized) or as described previously[1] in bulk format, or both (n=16 for each condition). Shown is the percentage (%) of reads with conversions. The line in the boxplot indicates the median value, the two hinges show the first and third quartiles. The whiskers range from the hinges to the highest or lowest point that is no further than 1.5 times the interquartile range. **(b)** Verification of T-C conversions in 4sU-labeld control RNA. Spike-in RNA was generated either with (4sU spike-in RNA) or without 4sU addition (non-4sU spike-in RNA). Shown are % T-C conversions. **(c)** Numbers of genes detected per cell for total single-cell RNA-seq data from K562 cells labelled with increasing concentrations of 4sU for 180 minutes labelling time. At least one read has to map to a gene for it to be counted as detected. The colours indicate two biological replicates. Median, hinges and whiskers shown as in **a**. **(d)** Representative genome browser tracks of the *MYC* gene of three 4sU-labelled and three unlabelled K562 cells showing coverage from all reads (grey) and coverage from reads with at least one or more T-C mutations (red, darker colour indicates higher number of T-C mutations, see legend). **(e)** Distribution of fraction of new RNA for genes with low (bottom 20%, n = 2374, left) and high (top 20%, n = 2374, right) turnover rates. Turnover rate was defined as synthesis rate * decay rate using rates estimated from bulk TT-seq data[2]. Only genes with a total read sum over all cells of at least 100 in NASC-seq data were considered. The *P*-value (<2.2e-16) was derived by two-sided Mann–Whitney U-test. Box limits are the first and third quartiles, the band inside the box is the median. The ends of the whiskers extend the box by 1.5 times the interquartile range. The source data for panels **a**, **c** and **e** are provided as a Source Data file.

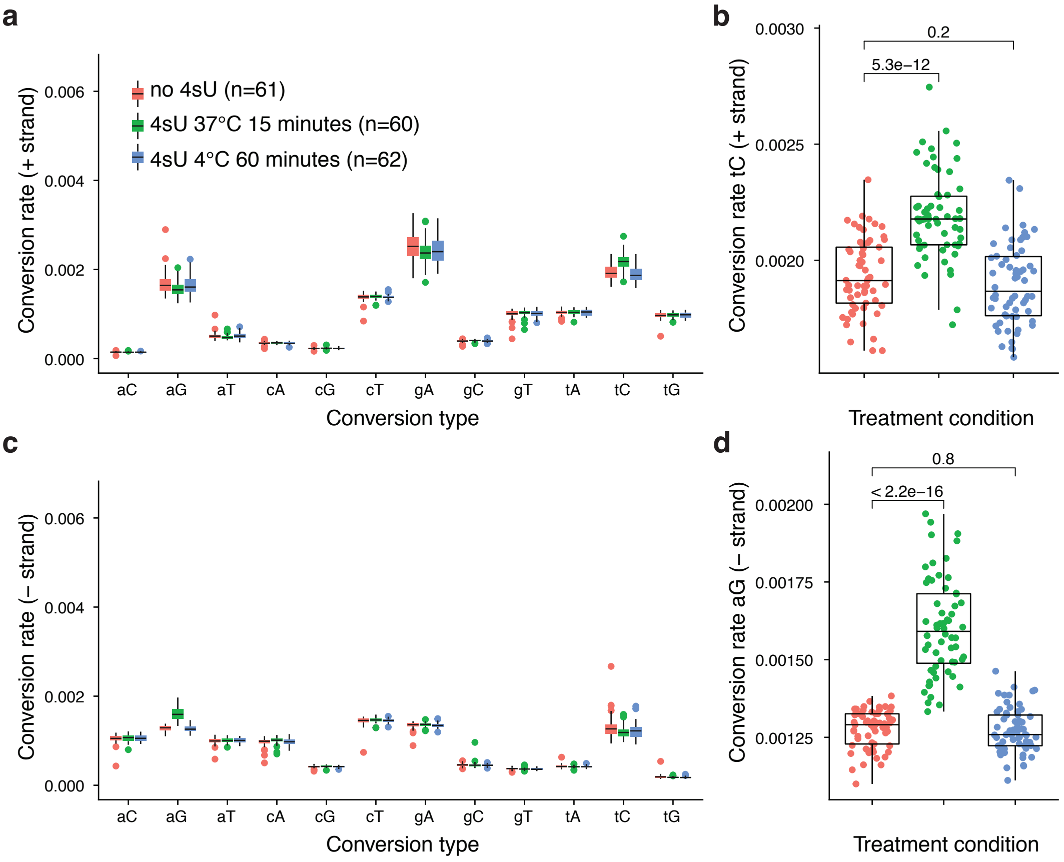

Supplementary Figure 2. Successful inhibition of label integration.
(a) Conversion rates for features on the positive strand for unlabelled cells, cells that were incubated with 4sU for 15 minutes at 37°C and cells that were incubated with 4sU on ice for 60 minutes. Lowercase and uppercase letters indicate original and new base identities, respectively. Line shows the median, first and third interquartile range is shown by hinges. The whisker extend up to 1.5 times the interquartile range. Points show outliers. **(b)** Boxplot with jittered visualization of individual data points showing T-C conversion rates for features on the positive strand. *P*-values shown were calculated by two-sided Mann-Whitney U test. Median, hinges and whiskers as in **a**. **(c)** Conversion rates for features on the negative strand for unlabelled cells, cells that were incubated with 4sU for 15 minutes at 37°C and cells that were incubated with 4sU on ice for 60 minutes. Median, hinges, whiskers and outlier points as in **a**. **(d)** Boxplot with jittered visualization of individual data points showing aG conversion rates for features on the negative strand. *P*-values shown were calculated by two-sided Mann-Whitney U test. Median, hinges and whiskers as in **a**. Source data are provided as a Source Data file.

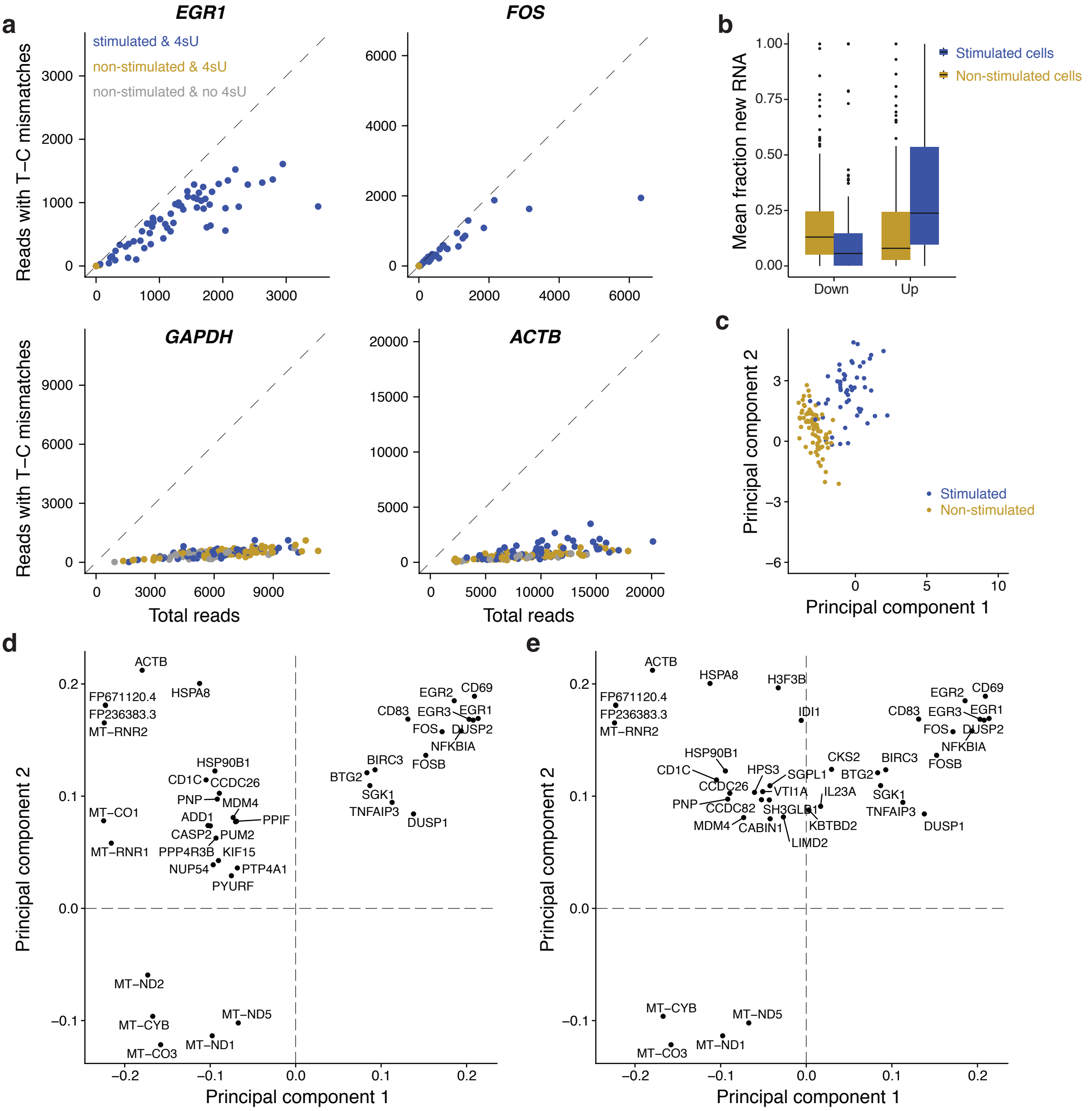

Supplementary Figure 3. Extended NASC-seq analysis of T-cell activation.

(a) Scatter plots showing the total number of sequenced reads (x-axis) against the number of reads with T-C conversions (y-axis) for *EGR1*, *FOS*, *ACTB* and *GAPDH*. Points are coloured by stimulation and labelling conditions. Non-stimulated cells essentially do not express *EGR1* or *FOS*, resulting in their absence from the top two panels.Source data are provided as a Source Data file. **(b)** Distribution of mean fraction of new RNA for genes which are detected as significantly up-regulated (right, n = 207) and down-regulated (left, n = 158) more than 2-fold upon T-cell stimulation (for 30 min with PMA and ionomycin) in newly synthesized RNA but not total RNA taken from a TT-seq experiment (see Methods). *P*-values (down-regulated genes = 5.845e-07, up-regulated genes = < 2.2e-16) were derived by two-sided Mann–Whitney U-test. Box limits are the first and third quartiles, the band inside the box is the median. The ends of the whiskers extend the box by 1.5 times the interquartile range. **(c)** Principal component analysis (PCA) for total RNA from Jurkat cells stimulated with PMA and ionomycin, and unstimulated cells. Axes are scaled to match axes in Figure 2d. **(d)** Gene loadings for the top 40 genes contributing to principal component 1 for principal component analysis shown in **c** and Figure 2d. **(e)** Gene loadings for the top 40 genes contributing to principal component 2 for principal component analysis shown in **c** and Figure 2d.

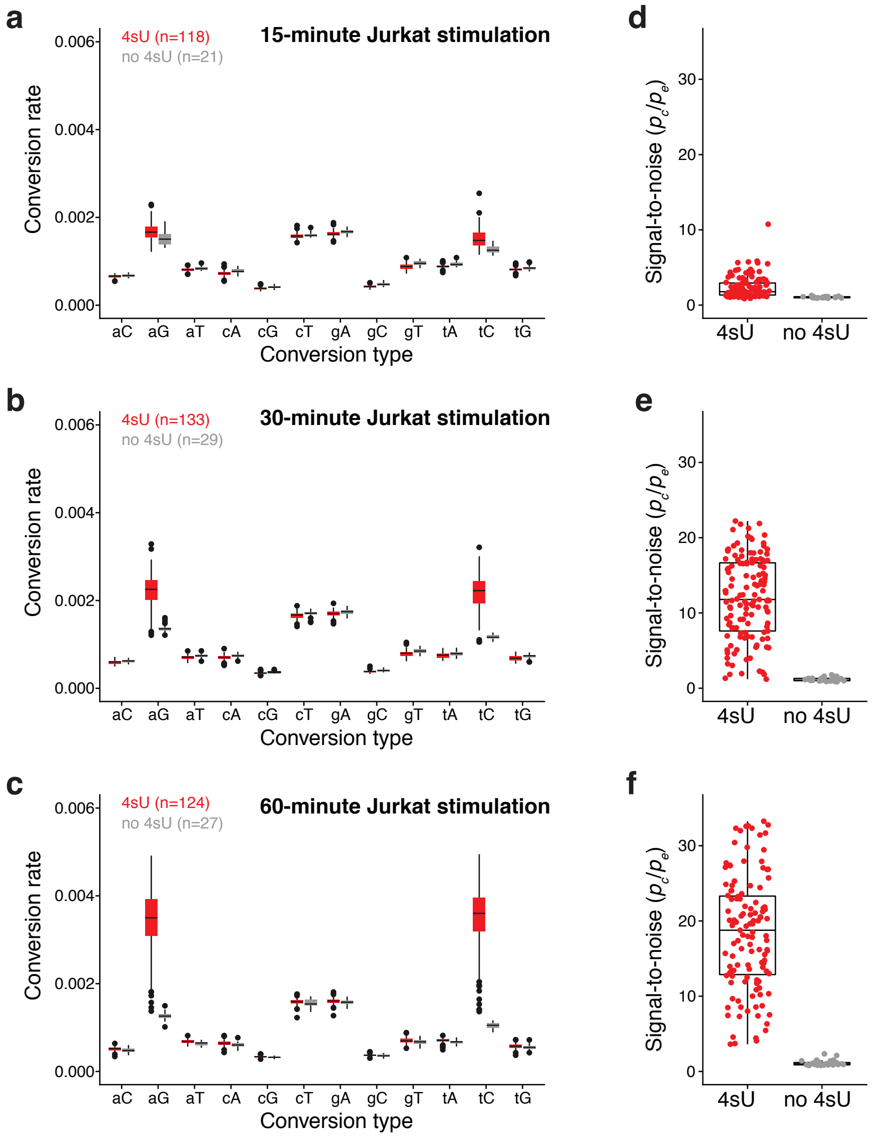

Supplementary Figure 4. Conversion rates Jurkat stimulation experiments.
Boxplots showing conversion rates for unlabelled and **(a)** 15-minute 4sU-labelled Jurkat cells, **(b)** 30-minute 4sU-labelled Jurkat cells and **(c)** 60-minute 4sU-labelled Jurkat cells. Lowercase and uppercase letters indicate original and new base identities, respectively. Signal-to-noise ratios for unlabelled and **(d)** 15-minute 4sU-labelled Jurkat cells, **(e)** 30-minute 4sU-labelled Jurkat cells and **(f)** 60-minute 4sU-labelled Jurkat cells. The lines in the boxplots indicate the median values, the hinges display the first and third quartiles. The whiskers range from the hinges to the highest or lowest point that is no further than 1.5 times the interquartile range. Source data are provided as a Source Data file.

Supplementary references

1. Herzog VA*, et al.* Thiol-linked alkylation of RNA to assess expression dynamics. *Nature methods* **14**, 1198-1204 (2017).

2. Schwalb B*, et al.* TT-seq maps the human transient transcriptome. *Science* **352**, 1225-1228 (2016).
